# Manipulating the strength of organism–environment feedback increases nonlinearity and apparent hysteresis of ecosystem response to environmental change

**DOI:** 10.1002/ece3.6294

**Published:** 2020-05-11

**Authors:** Aurélie Garnier, Florence D. Hulot, Owen L. Petchey

**Affiliations:** ^1^ URPP Global Change and Biodiversity University of Zurich Zürich Switzerland; ^2^ Institute for Marine Ecosystem and Fisheries Science University of Hamburg Hamburg Germany; ^3^ Université Paris‐Saclay CNRS, AgroParisTech Ecologie Systématique et Evolution Orsay France; ^4^ Department of Evolutionary Biology and Environmental Studies University of Zurich Zürich Switzerland

**Keywords:** dissolved oxygen, environmental change, feedback strength, hysteresis, nonlinearity, organism–environment feedback, stability, transient state

## Abstract

Theory predicts that organism–environment feedbacks play a central role in how ecological communities respond to environmental change. Strong feedback causes greater nonlinearity between environmental change and ecosystem state, increases the likelihood of hysteresis in response to environmental change, and augments the possibility of alternative stable regimes. To illustrate these predictions and their dependence on a temporal scale, we simulated a minimal ecosystem model. To test the predictions, we manipulated the feedback strength between the metabolism and the dissolved oxygen concentration in an aquatic heterotrophic tri‐trophic community in microecosystems. The manipulation consisted of five levels, ranging from low to high feedback strength by altering the oxygen diffusivity: free gas exchange between the microcosm atmosphere and the external air (metabolism not strongly affecting environmental oxygen), with the regular addition of 200, 100, or 50 ml of air and no gas exchange. To test for nonlinearity and hysteresis in response to environmental change, all microecosystems experienced a gradual temperature increase from 15 to 25°C and then back to 15°C. We regularly measured the dissolved oxygen concentration, total biomass, and species abundance. Nonlinearity and hysteresis were higher in treatments with stronger organism–environment feedbacks. There was no evidence that stronger feedback increased the number of observed ecosystem states. These empirical results are in broad agreement with the theory that stronger feedback increases nonlinearity and hysteresis. They therefore represent one of the first direct empirical tests of the importance of feedback strength. However, we discuss several limitations of the study, which weaken confidence in this interpretation. Research demonstrating the causal effects of feedback strength on ecosystem responses to environmental change should be placed at the core of efforts to plan for sustainable ecosystems.

## Graphical abstract

The organism–environment feedbacks play a central role in how ecological community response to environmental change. We simulated the “minimal ecosystem model” and incorporated different rates of environmental change to establish new predictions on the observation of nonlinearity and hysteresis. We confirmed those predictions using an experiment that manipulated the metabolism‐dissolved oxygen concentration feedback strength in response to a gradual change of temperature.

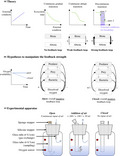

## 1 INTRODUCTION

Worldwide, all ecosystems are currently experiencing changing environmental conditions that may alter the feedbacks between biota and their environment (Naiman, Elliott, Helfield, & O’Keefe, 1999). Links in food webs can disappear (e.g., predator loss due to overexploitation: Estes et al., 2011), while fluxes can be altered, thus affecting the ecosystem state (e.g., eutrophication with nutrients in excess: Scheffer et al., 2003). The effects of climate change on ecosystems can have both top‐down and bottom‐up influences, and their feedbacks can be both positive and negative (Bony et al., 2006; Heimann & Reichstein, 2008; Moorcroft, 2003). Hence, the study of feedbacks and their consequences is key to understanding how ecosystems respond to environmental changes and management measures (Suding, Gross, & Houseman, 2004).

Organism–environment feedback (OEF) occurs when the activities of organisms affect their environment, and the environment simultaneously affects the organisms (Hutchinson, 1954; Jones, Lawton, & Shachak, 1994; Naiman et al., 1999; Tilman, 1988). In aquatic ecosystems, for example, organism respiration reduces the amount of dissolved oxygen (DO), while the amount of DO affects the vital rates of organisms (Breitburg, Loher, Pacey, & Gerstein, 1997; Fenchel, 2005; Fenchel & Finlay, 2008; Forster, Hirst, & Atkinson, 2012). Theoretical studies highlight the importance of OEF for diverse ecological issues such as niche construction (Jiang & DeAngelis, 2013; Odling‐Smee, Douglas, Palkovacs, Feldman, & Laland, 2013), competition (Golubski, 2007), population extinction (Qin, Zhang, Wang, & Song, 2017), metapopulations (Han, Hui, & Zhang, 2009), community structure (Muthukrishnan, Lloyd‐Smith, & Fong, 2016; Seto & Iwasa, 2011), and food web dynamics (Brown, Ferris, Fu, & Plant, 2004a).

**FIGURE 1:**
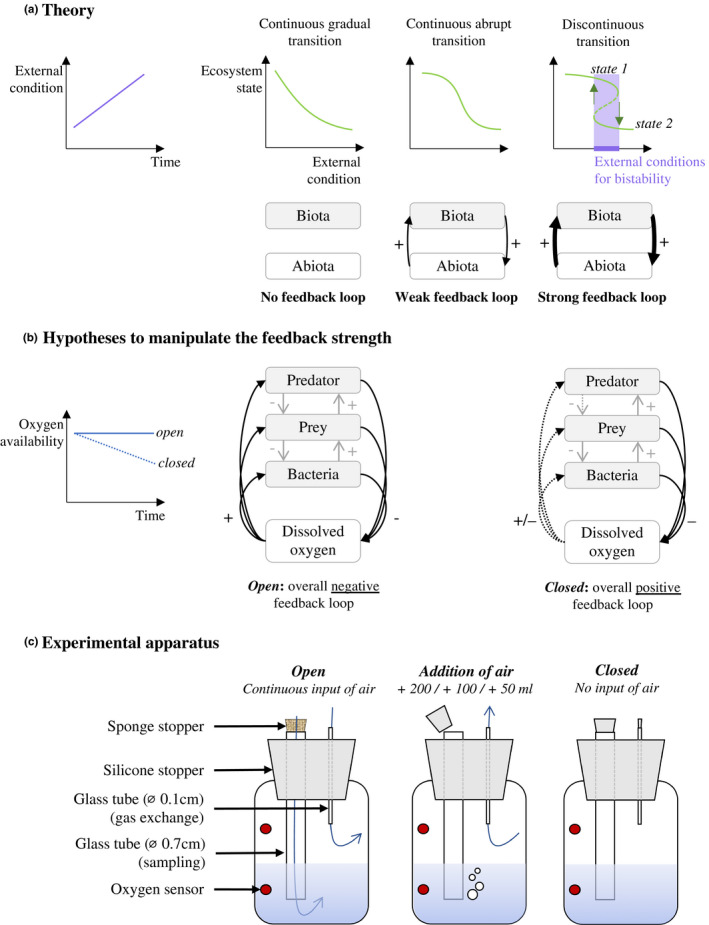
Theory, hypotheses, and experimental apparatus. (a) Three types of ecosystem responses to a gradual environmental change, depending on the feedback strength between biotic and abiotic compartments: in the absence of feedback, the ecosystem state will respond gradually, whereas the response will be highly nonlinear for a positive feedback loop. As the strength of the feedback increases, the ecosystem response becomes highly nonlinear with a catastrophic shift from *state 1* to *state 2* at a certain environmental condition threshold (represented by the green arrows). The environmental condition must decrease below the value at which the shift occurred to return to the previous state. Between these two environmental conditions, the system presents alternative stable states (i.e., bistability). (b) Manipulating the organism–environment feedback strength. The tri‐trophic system is composed of two negative feedback loops governed by predation (gray arrow). The biotic–abiotic feedback loop between the community and the oxygen availability is represented by positive and negative effects (black arrow). In an *open system*, the oxygen availability remains constant over time, with the consumed oxygen being replaced by gas diffusion from the atmosphere. By contrast, in a *closed system*, there is no supply, meaning that the available oxygen decreases over time. With a low oxygen concentration, the metabolic rate may reduce or increase (dotted arrow) depending on the organism response, resulting in a change in the overall feedback loop sign (e.g., from negative to positive, thus increasing the chance of alternative states). (c) Apparatus to manipulate the organism–environment feedback strength involving oxygen. The gas exchange manipulation consisted of blocking the glass tubes with silicone stoppers (addition of air and closed treatments) or with a sponge stopper (open treatment). The addition of air involved sucking 200, 100, or 50 ml of air via the small glass tube that bubbled the liquid phase. Oxygen sensors were used in each phase (head and liquid) to observe the oxygen concentration over time

OEFs have sparked the interest of ecologists as a potential determinant of ecosystem stability (Kéfi, Holmgren, & Scheffer, 2016; Lotka, 1925; Neutel, Heesterbeek, & Ruiter, 2002; Watson & Lovelock, 1983; Wilson & Agnew, 1992). Negative feedback loops tend to stabilize dynamics by dampening fluctuations that result from interactions, whereas positive ones can destabilize dynamics as fluctuations increase in magnitude (Jones et al., 1994; Kéfi et al., 2016; Lenton, 2013; Lewontin, 1969; Marzloff, Dambacher, Johnson, Little, & Frusher, 2011; May, 1977; Scheffer, Carpenter, Foley, Folke, & Walker, 2001; Seto & Iwasa, 2011). The balance of negative and positive feedbacks is predicted to control the linearity of the response of an ecosystem to environmental change and determine whether that response exhibits hysteresis and abrupt changes (i.e., tipping points) (Figure 1a).

A well‐known example of a nonlinear and hysteretic response of an ecosystem to environmental change is found in shallow lakes, in which the ecosystem state is mainly governed by OEF loops involving aquatic vegetation and phytoplankton (Scheffer, 2004; Scheffer & van Nes, 2007). Two stable states can be observed depending on the nutrient concentrations. With low nutrients, the only observed state is dominated by macrophytes and corresponds to clear water: macrophytes deplete the nutrients, thus negatively impacting the phytoplankton and promoting water transparency, which in turn has a positive effect on macrophytes. Macrophytes also provide refuge to zooplankton, which can control phytoplankton abundance. With high nutrients, the other stable state is observed, characterized by turbid water. Enrichment promotes phytoplankton growth, which affects the environment by increasing suspended particles, decreasing light availability, and reducing macrophytes and thus zooplankton refuges. These two states are each stable due to feedback loops previously described (Scheffer et al., 2001). At intermediate nutrient levels, either of these states can be observed depending on the historical conditions; in other words, there are two alternative stable states.

The turbid state is often considered an undesirable state, and so measures such as reducing nutrient loading are sometimes taken to recover the more desirable clear water state (Meijer, Raat, & Doef, 1989; Scheffer et al., 2001). However, the measures may be unsuccessful, with the shallow lake remaining turbid after a reduction in nutrient loading (Meijer et al., 1989). The lake will only recover to a clear state after a substantial decrease in nutrient concentrations, at a level below which the lake initially switched to the turbid state. This phenomenon, termed hysteresis, refers to the historical dependency of the system state (Holling, 1973; May, 1977). In general, and as mentioned, feedback systems theory predicts that stronger positive feedback can increase the likelihood of hysteresis and nonlinearity (Cinquin & Demongeot, 2002; Lenton, 2013; Kéfi et al., 2016; van de Leemput, Hughes, van Nes, & Scheffer, 2016; Figure 1a).

These types of systems dynamics and the role of feedbacks in inducing them may be observed in other systems such as deserts, coral reefs, woodlands, and oceans, where other biological and environmental factors are involved in the feedback loop such as fishing, overgrazing, and climate change (Riegl & Piller, 2000; Scheffer et al., 2001). In the North Sea, for example, the critical transition between cod‐ and herring‐dominated states had a profound effect on the local economy. The environmental factor behind this critical transition was likely the increasing sea surface temperature, which favored herring population growth while harming cod recruitment (Beaugrand, Brander, Lindley, Souissi, & Reid, 2003; O'Brien, Fox, Planque, & Casey, 2000). Hence, gradually changing temperatures may be one of the greatest challenges for ecosystem management under climate change.

Numerous studies have tested for the presence of multiple stable states using various types of evidence (Dai, Vorselen, Korolev, & Gore, 2012; Fukami, Bezemer, Mortimer, & Putten, 2005; Jiang & Patel, 2008; Louette & De Meester, 2007; Sait, Liu, Thompson, Godfray, & Begon, 2000; Schröder, Persson, & Roos, 2005, 2012; Weslien, Djupström, Schroeder, & Widenfalk, 2011). However, given the importance of the feedback strength in governing system response to environmental change (Ratajczak et al., 2018), it is somewhat surprising that—to our knowledge—no experimental studies have manipulated OEF strength and tested its effects on nonlinearity, hysteresis, and multistability (Schröder, Persson, & Roos, 2005). Experimental manipulations of the chemostat dilution rate come close, as the dilution rate influences the effect of resource consumption on resource concentration (Dai et al., 2012; Fussmann, Ellner, Shertzer, & Hairston, 2000). A study by Dai et al. (2012) involved yeast populations in chemostats that exhibited an Allee effect (positive density‐dependent growth at a low population size), alternative stable states, and hysteresis. However, the experiment did not aim to test the prediction that a stronger/weaker Allee effect would increase/decrease the likelihood of alternative stable states, hysteresis, and nonlinear response to environmental change.

Our study aimed to examine the effects of OEF strength on ecosystem dynamics and ecosystem response to environmental change (temperature). Small laboratory‐based communities of aquatic microorganisms are a relevant and convenient study system, given the diversity of common ecological processes (growth, death, consumption, competition, predation), the fast generation time of the organisms, and their ease of monitoring and manipulation. Our community was a predator–prey–resource system and thus had negative feedback loops between species abundances. The organisms involved perhaps show different non‐monotonic responses to DO, such that a reduction in DO could be associated with increased metabolism (i.e., positive feedback) (especially for anaerobic organisms: e.g., Fenchel, 2005; Hardewig, Addink, Grieshaber, Pörtner, & Thillart, 1991), with low DO being associated with reduced rates such as the predation rate if the predator is intolerant to anoxia (Decker, Breitburg, & Purcell, 2004; Nestlerode & Diaz, 1998; Sandberg, Tallqvist, & Bonsdorff, 1996). DO concentration was the main environmental variable, since it is a key component in aquatic ecosystems and depends on physical and biological processes. We conceived a method for manipulating the strength of OEF using oxygen by altering the openness of the microcosms to the surrounding atmospheric gases (Figure 1c). When open to the surrounding atmosphere, the oxygen consumption of organisms has a weaker effect on DO, since the used oxygen can quickly be replaced from the surrounding atmosphere. When closed, consumption has a stronger effect, thus reducing DO concentration. By manipulating the openness of the microcosm to the surrounding atmosphere, we manipulated the strength of the feedback loop between organisms and their environment, with the possibility of creating stronger positive feedback in the ecosystem. If the manipulation of the oxygen availability leads to this effect, then the theory predicts that increasing the feedback strength will: (a) cause greater nonlinearity between an environmental change and ecosystem state; (b) increase the likelihood of hysteresis in response to an environmental change; and (c) augment the possibility of observing alternative stable states (Fussmann et al., 2000; Ibelings et al., 2007; Kéfi et al., 2016; Rietkerk, Dekker, Ruiter, & Koppel, 2004; Scheffer & Carpenter, 2003).

However, this manipulation may simultaneously affect the rate at which the DO of the system changes over time (Figure 1b) due to the different rates at which oxygen is replenished. Indeed, a high biomass community in a closed system could result in a faster DO decrease compared to a low biomass community (with lower oxygen consumption). Nevertheless, high and low biomass may not differ in an open system when the respiration is balanced with diffusion from the headspace (Figure 1b). This raises the possibility that changes in the rate of system responses to the environmental driver changes could also affect the results. We therefore needed to develop theoretical predictions contingent on this possibility. Hence, we first developed and described the predictions of the minimal ecosystem model presented in Scheffer et al. (2001). We then compared the experimental results to these predictions.

## 2 MATERIAL AND METHODS

### 2.1 Development of theory and predictions

Imagine an ecosystem state *Y* (e.g., DO concentration in a pond) and an environmental driver (e.g., temperature) in state *E.* When the environmental driver previously followed an increasing trend, we used *Y*(*E_up_*) to describe the ecosystem state, and when the environmental driver previously followed a downward trajectory, we used *Y*(*E*_down_).

The amount of nonlinearity was calculated as the root mean square difference between the fitted values of a linear and nonlinear model of the *Y* versus *E* relationship (Emancipator & Kroll, 1993). A generalized additive model fitted using the default options of the *gam* function in the *mgcv* r package was the nonlinear function (Wood, 2011), while the linear model was fitted using the *lm* function in the *stats* r package (R Core Team, 2016). As opposed to relative nonlinearity, response data were not standardized to obtain a measure of absolute nonlinearity (Emancipator & Kroll, 1993).

The amount of hysteresis was measured as the difference between *Y*(*E*_up_) and *Y*(*E*_down_) for all *E*; we used the mean of the absolute difference. The absence of hysteresis implies that the ecosystem state will follow the same trajectory (see Figure 1a—continuous vs. discontinuous transition) when the environmental driver increases or decreases such that *Y*(*E*_up_) = *Y*(*E*_down_). Hysteresis implies that the path of the ecosystem state differs depending on the direction of environmental change such that *Y*(*E*_up_) ≠ *Y*(*E*_down_).

To simulate changes in the ecosystem state caused by changes in an environmental driver, we used the “minimal ecosystem model” presented in Scheffer et al. (2001):

$$\frac{\mathrm{dY}}{\mathrm{dt}}=a-bY+rf\left(Y\right)\;\text{and}\;f\left(Y\right)=\frac{Y^{p}}{Y^{p}+h^{p}}$$

where *Y* is an ecosystem property, *a* an environmental driver that promotes *Y*, *b* the rate of decay of *Y*, *r* the “self‐replacement” rate of *Y* that is modified by the function *f(Y)* in which *p* controls the nonlinearity of this self‐replacement function (i.e., feedback strength), and *h* the threshold at which the shift occurs. We set *b* = *r* = *h* = 1. We then simulated the system with *p* (i.e., feedback strength) set at values ranging from 0 to 10, and *a* changing linearly from 0 to 1 and back to 0 at each of the two rates of change: up‐and‐down in 200 timesteps (fast rate of change) or 20 000 timesteps (slow rate). For each combination of *p* and rate of change of *a*, we checked for alternative stable states and measured nonlinearity and hysteresis, as described above. We also made the same simulations but with decreasing and then increasing changes in *a*. A reproducible description of this simulation study is available online at http://opetchey.github.io/RREEBES/Scheffer_etal_2001_Nature/report.html.

### 2.2 Experimental system

Microcosms were sterile 250 ml glass jars containing 100 ml protozoan pellet medium (PPM) (Altermatt et al., 2015). Media consisted of 0.55 g of crushed protozoan pellets (Carolina Biological Supply Co., Burlington, N.C. USA) in 1 L of Chalkley's medium and then filtered through a sterile 0.45 µm membrane filter before sterilization by autoclave. Filtration was used to remove small particles to avoid debris affecting the videography and flow cytometry without affecting dynamics (unpublished work). Two additional wheat seeds per microcosm provided a slow‐release nutrient source. Microcosms were placed in a dark temperature‐controlled incubator to reduce the possibility of unwanted photosynthetic organisms.

The microbial heterotrophic aquatic community consisted of two bacteria species (*Serratia fonticola* and *Bacillus subtilis*), two bacterivorous prey species (*Colpidium striatum* and *Dexiostoma campylum*), and one predator species (*Spathidium* sp.). To avoid extinction caused by starvation, *Spathidium* sp. forms cysts and emerges when prey increase in abundance. We initiated the community with bacteria grown at 37°C for 24 hr and then added *Colpidium striatum* and *Dexiostoma campylum*. Before this addition, the prey species were grown in monoculture for 7 days at 15°C to reach carrying capacity. On day 0, in the microcosms we combined 45 ml of *Colpidium striatum's* culture (with ~100 ind.mL^−1^), 45 ml of *Dexiostoma campylum's* culture (with ~300 ind.mL^−1^), and 10 ml of *Spathidium* sp. at a density of 12 individuals per mL.

### 2.3 Experimental design

Measuring the nonlinearity of responses to an environmental driver requires the environmental driver to be varied. Measuring hysteresis requires the variation in an environmental driver to occur in two directions. Neither measurement requires a controlled environmental treatment for comparative purposes (see our simulation results below and Schröder et al., 2005). Therefore, we exposed all microcosms to the same temperature regime: an increase of 0.7°C every two days for 30 days, constant at 25°C for a week, and then a decrease of 0.7°C every two days for 30 days. This rate (+2.5°C per week or about 0.1–0.2°C per generation) is comparable to the predicted temperature rise over the next 100 years that will affect larger organisms (IPCC, 2007). Additionally, this temperature range was relevant given the wide thermal tolerance of protists (Atkinson, Ciotti, & Montagnes, 2003; van der Have & de Jong, 1996; Krenek, Berendonk, & Petzoldt, 2011; Laakso, Löytynoja, & Kaitala, 2003). Monocultures were kept at 15°C in their long‐term stock culture (Altermatt et al., 2015). Note that we simulated two rates of environmental change in the model, while we tested only one rate of environmental change in the experiment.

**FIGURE 2:**
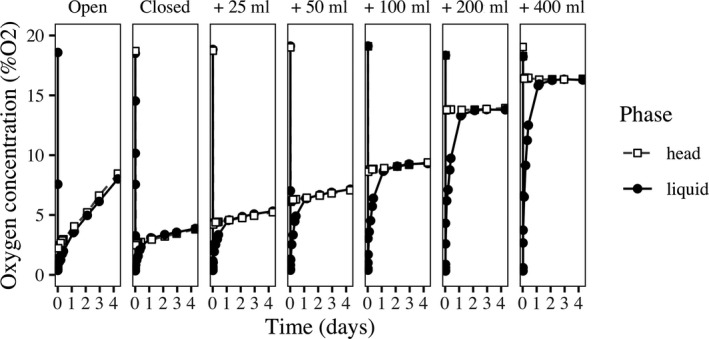
Gas exchange manipulation method. Firstly, oxygen was removed from the water using nitrogen gas (N_2_). Oxygen was then recovered in the liquid (black circles) and head phases (white squares)

To manipulate the strength of the OEF loop, we altered the strength of the effect of organism respiration on DO concentration. We varied the rate of gas exchange between the atmosphere surrounding the microcosms (assumed constant at 21% oxygen) and the headspace of the microcosm (Figure 2). With a higher rate of gas exchange (i.e., open system), the effects of organism respiration (i.e., oxygen consumption) on DO concentration would be weaker, as the consumed oxygen would be replaced by oxygen diffusion. This would lead to a weaker feedback loop (Figure 1b). However, a lower rate of gas exchange (i.e., closed system) would lead to stronger effects of respiration on DO (and a stronger feedback loop; Figure 1b), as the respiration is not balanced by the diffusion. In preliminary experiments, we observed that DO was more strongly affected by metabolism when the jars were more closed (unpublished data). Nevertheless, this does not necessarily make oxygen a limiting resource, as this depends on absolute DO levels rather than feedback strength.

The rate of gas exchange was controlled by sealing each microcosm jar with a 3‐cm silicone stopper with two holes (Figure 1c). The first hole contained a glass tube with a 0.7 cm inside diameter to allow microcosm sampling with a Pasteur pipette; it was sealed with a 0.7‐cm silicone stopper (to prevent gas flux) or a sponge (to allow gas flux). The second hole contained a hypodermic needle with a 0.1‐mm inside diameter fitted with a plastic adaptor that was sealed with a 0.4‐cm silicone stopper. This apparatus allowed us to implement five levels of oxygen exchange treatment: continuous exchange (by putting a sponge in the glass tube), exchange of 50, 100, or 200 ml air every second day (via the needle, and otherwise with a stopper in the glass tube and hypodermic needle), or very little exchange (a stopper in the glass tube and hypodermic needle). The experiment resulted in 30 microcosms with five oxygen exchange levels and six replicates of each. A small amount of air was exchanged every time the communities were sampled; hence, all the treatments, even the one with the stopper, were not completely sealed to gas flux. The quantified gas exchange treatments (addition of 50, 100, or 200 ml air) were carried out by sucking the air through the 0.1‐mm needle with a syringe that created a suction effect and added air directly in the liquid phase. Test experiments validated that these treatment levels had the expected effects on DO concentrations (Figure 2).

### 2.4 Data collection

All measurements were made every 2 days.

#### 2.4.1 Oxygen measurement

The percentage of oxygen was measured using noninvasive chemical‐optical sensing (Fibox 4trace, PreSens, Germany; Altermatt et al., 2015). This method involved fixing sensors to the inside of the microcosm vessel walls in the head and liquid spaces (at the same depth in all microcosms). These were read by a reader machine with a fiber optic cable to read the sensors detecting the oxygen level and a temperature probe to record the temperature of the microcosm. The probes function equally well when wet or dry. The oxygen sensors were precalibrated by the PreSens company and checked in water at 20°C before the experiment. The sensor reader calculates %O_2_ in the liquid and headspace of the microcosms while adjusting to the temperature of the microcosm. Hence, the reader reports 21%O_2_ when the liquid and headspace gas are balanced, regardless of the temperature of the liquid and headspace gas. At each sampling event, oxygen was first measured without moving the microcosms to avoid any effects of movement.

#### 2.4.2 Predator density

After gently agitating the microcosm for the purpose of homogenization, we sampled 1 ml with a Pasteur pipette and replaced it with fresh PPM. We estimated the predator density by counting the number of *Spathidium* sp. individuals in 1 ml by eye under a dissecting microscope.

#### 2.4.3 Prey density with video analysis

To estimate the prey density, we used video analysis (Pennekamp, Schtickzelle, & Petchey, 2015). In a custom counting chamber, we placed 700 µl of the 1 ml previously used to count the predators and made a 5‐s video at 25 frames per second of ~50 µl of the 700 µl using a camera (Hamamatsu Digital camera C11440) attached to a microscope (Leica M205C, 0.63X) and relevant software (HCImage Live version 4.0.6.3). The videos were analyzed using the BEMOVI package; this software isolates moving particles (here, ciliates), reconstructs their trajectories, and assigns trajectories to species based on morphological traits (Pennekamp et al., 2017). The customized counting chamber had a 0.6‐mm depth (compared to 1 mm for a Sedgewick rafter slide) to reduce the vertical movement of individuals during the video measurement and therefore increase the accuracy of measured morphological traits.

#### 2.4.4 Total biomass

Similarly to prey density, we used the morphological traits (width (*a*) and length (*b*)) of the individuals identified in the video to estimate the biovolume of the prey and predator populations while assuming an ellipsoid shape: $\text{biovolume}=\left(4/3\right)\cdot \pi \cdot {\left(a/2\right)}^{2}\cdot \left(b/2\right)$

#### 2.4.5 Bacteria density with a flow cytometer

We diluted 20 µl from the samples in 160 µl of filtered ultrapure water and 20 µl of a 10‐fold dilution of SYBR® Green. This mixture was incubated at 37°C for 15 min in the dark to stain the DNA in each cell. The flow cytometer (Accuri C6 with multi‐well sampler, BD Biosciences, San Jose, CA, USA) was run with the following parameters: sampled volume 30 µl; medium fluid speed; FSC‐H threshold of 20 000 and SSC‐H threshold of 400. These two parameters refer to morphological traits: forward scatter (FSC) is proportional to particle size, while side scatter (SSC) measures the cellular roughness or internal complexity. These thresholds allow for the detection of small bacteria such as *Serratia fonticola* (e.g., Griffiths, Petchey, Pennekamp, & Childs, 2018; Tabi, Petchey, & Pennekamp, 2019).

### 2.5 Measured variables

Firstly, we focused on DO concentration and total biomass (i.e., ecosystem states), as they summarize the state and effect of the ecological community. With these ecosystem variables, we characterized two components (nonlinearity and hysteresis) to describe how the ecosystem state changes with a gradual change in temperature.

#### 2.5.1 Nonlinearity

For each microcosm and separately for the temperature increase and decrease phases, we calculated the nonlinearity of the relationship between DO concentration and total biomass against the temperature. Nonlinearity was calculated as in the simulations.

#### 2.5.2 Hysteresis

To estimate hysteresis in response to the increasing and then decreasing temperature experienced by each microcosm, we compared the DO concentrations and total biomass measured at a given temperature. A close match between the ecosystem variable in the increasing and decreasing temperatures would indicate a lack of hysteresis, while a difference would indicate hysteresis. For each microcosm, we estimated hysteresis as in the simulations (i.e., mean absolute difference between the paired ecosystem variables).

### 2.6 Statistical analyses

Analyses were performed using the statistical software r (R Core Team, 2016).

#### 2.6.1 Time series analysis

We performed principal component analysis (*vegan* r package: Oksanen et al., 2019) to detect a temporal change in community (predator, prey, and bacteria density) and ecosystem variables (DO concentration and total biomass) over time. Species abundances were log10 transformed; all variables (species abundance, DO and total biomass) were scaled to obtain a standard deviation of 1 and centered (mean equal to 0) to enable comparison. We also performed cluster analysis to detect the presence of potentially alternative regimes based on the (dis)similarity between time series. Indeed, two alternative regimes would be characterized by two dissimilar dynamics. (Dis)similarity was measured using a dynamic time warping (DTW) distance (Sarda‐Espinosa, 2019). The number of alternative regimes was assessed using hierarchical cluster analysis on this DTW distance with the Ward agglomeration method. This analysis was performed with the *dtwclust* r package (Sarda‐Espinosa, 2019). To search for evidence that the manipulation of feedback strength affected the presence and number of alternative regimes, we tested whether the cluster was associated with the gas exchange treatment using logistic regression and the likelihood ratio test (*stats* r package).

#### 2.6.2 Nonlinearity and hysteresis analyses

To test for an effect of the gas exchange treatment (i.e., feedback strength) on nonlinearity and hysteresis measurements, we used an analysis of variance (ANOVA) with the feedback strength treated as a categorical explanatory variable. We added the membership of the previously identified clusters as categorical explanatory variables, resulting in an analysis of covariance (ANCOVA).

## 3 RESULTS

### 3.1 Development of theory and predictions

**FIGURE 3:**
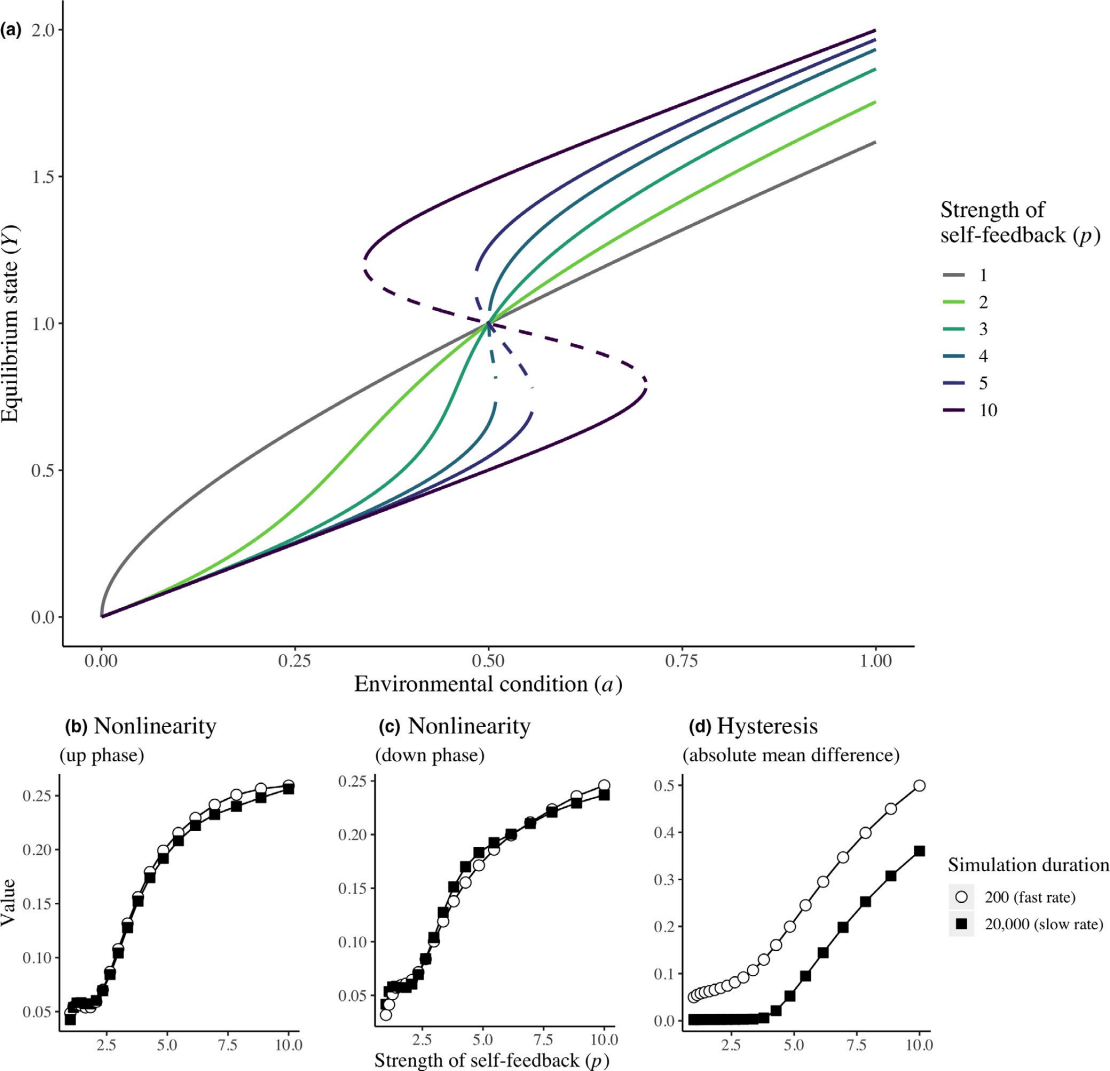
Simulation results using the minimal ecosystem model (Scheffer et al., 2001). (a) The ecosystem state shifts when increasing the environmental condition depending on the strength of the positive feedback (colors). (b–d) Simulation results showing the effects of the strength of positive feedback and the rate of environmental change on the empirical measures in (b‐c) nonlinearity and (d) hysteresis of response to an environmental driver. Alternative stable states are present only above x‐axis values of about 4

Let us first examine the case where *p*, nonlinearity in the self‐replacement term or feedback strength, is less than ~4, in which case there is no positive feedback in the system and only one stable state for any value of the environmental driver (Figure 3a). With a slow rate of environmental change, there is no measured hysteresis (as expected) and no effect of *p* on measured hysteresis. By contrast, with a fast rate of environmental change, hysteresis is observed, and an increase in *p* causes an increase in measured hysteresis (Figure 3d). Nevertheless, even with *p* < 4, nonlinearity rises with increases in *p* regardless of the rate of environmental change, although the general increase contains some stable or even slightly downward phases (Figure 2b,c).

Now consider when *p* > 4, such that there is positive feedback in the systems, with two stable states existing for some values of the environmental driver. Here, the measures of hysteresis and nonlinearity behave as expected, with increases in *p* leading to greater hysteresis and nonlinearity regardless of the rate of environmental change (Figure 3). The extent of hysteresis, however, is greater when environmental change is faster.

Results with the opposite pattern of environmental forcing (*a* decreasing and then increasing) differed only in terms of observed absolute nonlinearity during the downward phase. In this case, greater nonlinearity was observed when environmental change was faster.

### 3.2 Empirical results: Evidence for alternative dynamic regimes

**FIGURE 4:**
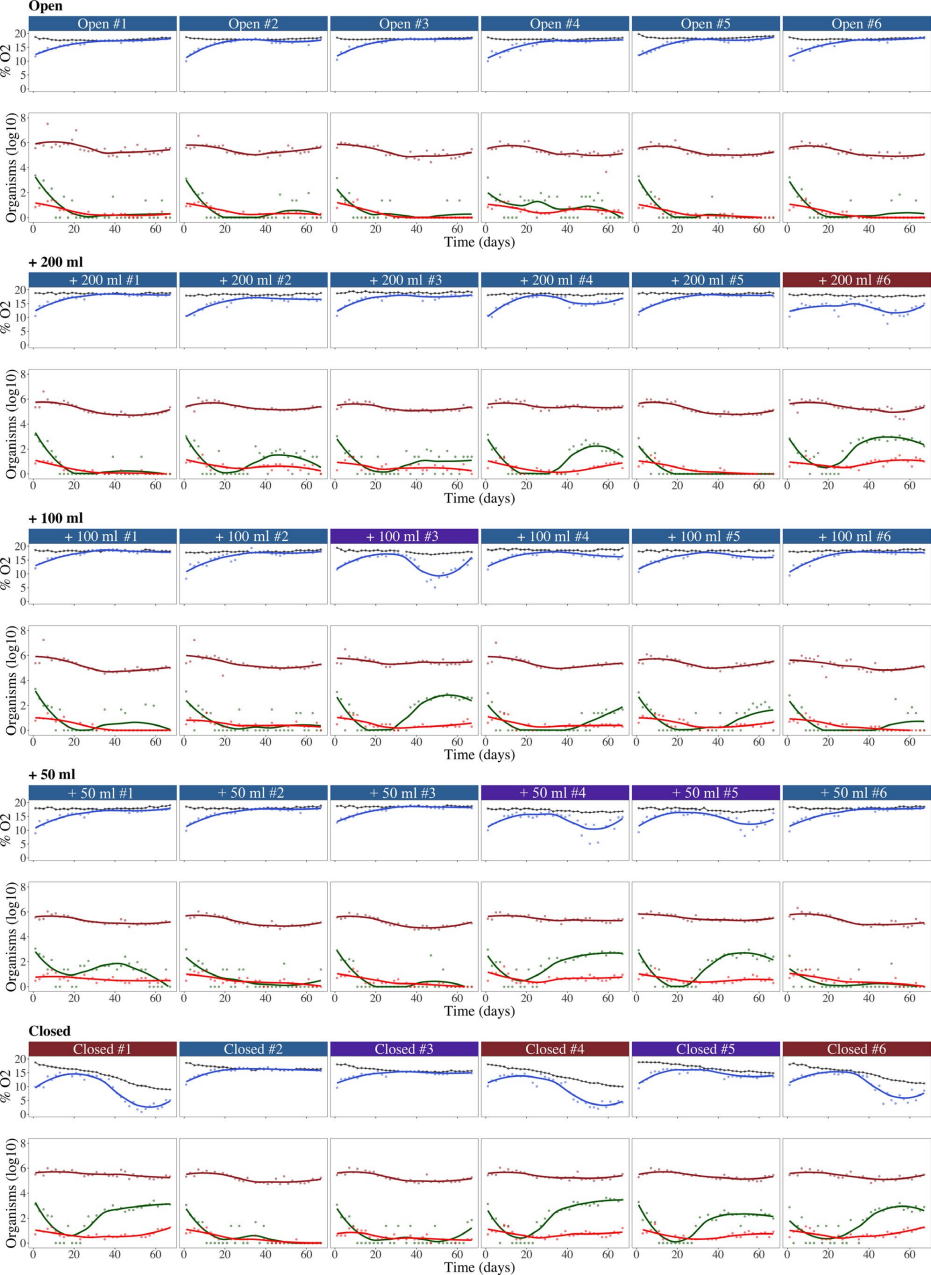
Dynamics of %O_2_ in the liquid (dark blue), headspace (black), bacteria density (brown), prey (green), and *Spathidium* sp. (red) for each microcosm (i.e., five gas exchange treatments with six replicates). The label colors correspond to the clusters in Figure 5b

**FIGURE 5:**
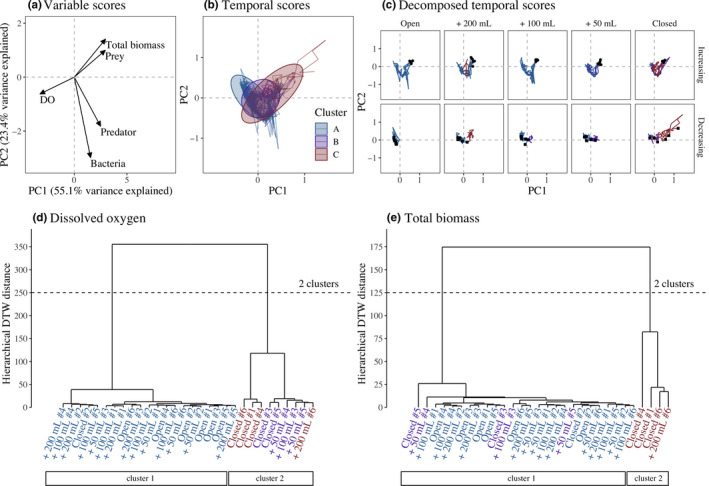
Analyses of community and ecosystem dynamics. The first two dimensions of principal component analysis (PCA) explained 78.5% of the variance observed with (a) the overall correlation between the variables and (b‐c) the temporal trend of the PCA scores. The temporal trend was divided into the gas exchange treatment and temperature phase to facilitate the reading, with the beginning (black circle) and end (black square) of the dynamics. The time series cluster analysis for (d) DO and (e) total biomass highlighted two clusters (i.e., two dynamic regimes). The clusters (A, B, and C) in panel (b) resulted from the combination of two cluster analyses (panels d and e) with cluster A (in blue) grouping the microcosms classified in both “cluster 1,” cluster B (in purple) grouping the microcosms in “cluster 2” for DO and in “cluster 1” for total biomass, and cluster C (in red) grouping the microcosms classified in both “cluster 2”

All communities started with around 10% DO, 10^6^ bacteria per ml, 1,000 prey per ml, and 10 predators per ml (Figure 4). In the principal component analysis (PCA) of variability in the ecosystems through time and among replicates (Figure 5), the first axis represented 55.1% of the variance, and generally, DO and organisms were negatively associated. The second axis (23.4%) represented a variation in a typical trophic cascade. For example, when the prey population was high (positive PC2 scores), the predator and bacterial populations were low (negative PC2 scores) (Figure 5a). Total biomass and prey density were highly correlated, as the latter made up a large part of the former.

The gas exchange treatment, especially the addition of air by bubbling, did not affect the nutrient concentrations, as there was no difference in the mean bacterial density (ANOVA, *F*_4,25_ = 1.36, *p* = .27). Therefore, for the remainder of the manuscript, we focus on predator–prey dynamics. During the increasing temperature phase (days 1–33), the dynamics were similar in all gas exchange treatments (Figure 5c) with a decrease in prey and an increase in predators. DO also increased with the diffusion from the headspace into the liquid. During the decreasing temperature phase (days 34–67), the dynamics started to diverge between and within the gas exchange treatments (Figure 5c). In the open system, the dynamics were constant over time (i.e., no change in the temporal scores) with low prey, low predators, and high DO concentration (negative PC1 scores; Figure 5c). In the other gas exchange treatments, the dynamics were less similar. Most replicates were in the same PCA space as the “open” treatment, whereas a few showed higher total biomass and/or lower DO concentration (i.e., positive PC1 scores). In the closed treatment, the divergence between replicates was larger than for the other gas exchange treatments.

The divergence in dynamics within treatments was confirmed with the time series cluster analysis. Two clusters described the dynamics of DO (Figure 5d) and total biomass (Figure 5e). For DO, the first cluster with 21 microcosms referred to the stabilized DO (negative PC1 scores). The second cluster with nine microcosms (none “Open”, one “+200 ml”, one “+100 ml”, two “+50 ml”, and five “Closed” replicates; Figure 5d) described the dynamics with lower DO values (positive PC1 scores).

Statistical evidence showed that a community in a particular cluster was influenced by the gas exchange treatment (logistic regression with likelihood ratio test (LRT): *df* = 4, deviance = 12.79, Pr(>Chi) = 0.012). For the total biomass, only four microcosms formed the second cluster (one “+200 ml” and three “Closed” replicates; Figure 5e). This cluster described dynamics with a larger total biomass (positive PC1 and PC2 scores; Figure 5b). Note that the microcosms identified in this cluster were also characterized by lower DO concentrations. The probability of having a microcosm from the “closed” system in this cluster was marginally significant (logistic regression with LRT: *df* = 4, deviance = 9.84, Pr(>Chi) = 0.043).

### 3.3 Empirical results: Nonlinearity of environmental change and system state relationship

For DO, during the gradual temperature increase, nonlinearity was greater in replicates with stronger feedback (i.e., those with less gas exchange) (Table 1; Figure 7a). During the gradual temperature decrease, there was a similar trend toward greater nonlinearity in more closed treatments, but without statistical significance (Table 1) due to the higher variability among replicates (Figure 7b).

**TABLE 1:** Statistical summary of the effect of gas exchange (i.e., feedback strength) on nonlinearity for the increasing and decreasing temperature phases as well as hysteresis for the measurements of dissolved oxygen and total biomass. We considered the gas exchange treatment to be a categorical variable (with ANOVA). Additionally, we tested the temporal dynamics depicted with hierarchical cluster analysis. Therefore, the responses were analyzed with ANCOVA in which the explanatory variables were the gas exchange and the cluster, both categorical variables. We tested the interaction between the gas exchange and the cluster, although we only reported the data when the difference between the models with and without the interaction was significant (*p* < .05)

	Dissolved oxygen				Total biomass			
	ANOVA		ANCOVA		ANOVA		ANCOVA	
	F	*p*	F	*p*	F	*p*	F	*p*
Nonlinearity during the increasing temperature phase								
Gas exchange	3.040	**.036**	3.125	**.033**	1.345	.281	1.293	.301
Cluster			1.702	.204			0.016	.899
Nonlinearity during the decreasing temperature phase								
Gas exchange	0.915	.471	3.325	**.027**	2.458	.072	4.389	**.009**
Cluster			66.815	**<.001**			13.910	**.001**
Interaction							7.733	**.011**
Hysteresis								
Gas exchange	2.115	.109	2.447	.074	2.789	**.048**	3.999	**.013**
Cluster			4.932	**.036**			11.849	**.002**

We observed that during the gradual temperature increase, nonlinearity did not differ between the two clusters (Table 1). Indeed, the DO in all the microcosms increased toward the oxygen level in the head phase (Figure 4), while the difference in nonlinearity observed between treatments was due to a difference in the level reached. Interestingly, the nonlinear response of DO to decreasing temperatures depended on the cluster of a given community (Table 1; Figure 7b). When the predator–prey system persisted, the consumption of oxygen was greater than the supply, leading to a decrease in DO. This pattern was especially pronounced in the closed systems for the three microcosms. However, in microcosms with extinctions or in open systems, oxygen levels remained stable (i.e., linear) over the temperature decrease due to the absence of consumption or its compensation by diffusion.

For the total biomass, the feedback strength had no effect in terms of increasing temperature (Figure 7d), regardless of whether the cluster was included or not (Table 1). However, the nonlinear response of the total biomass to decreasing temperatures depended on the cluster of a given community. This pattern was especially pronounced in the closed systems for the three microcosms (Table 1; Figure 7e).

### 3.4 Empirical results: Hysteresis

**FIGURE 6:**
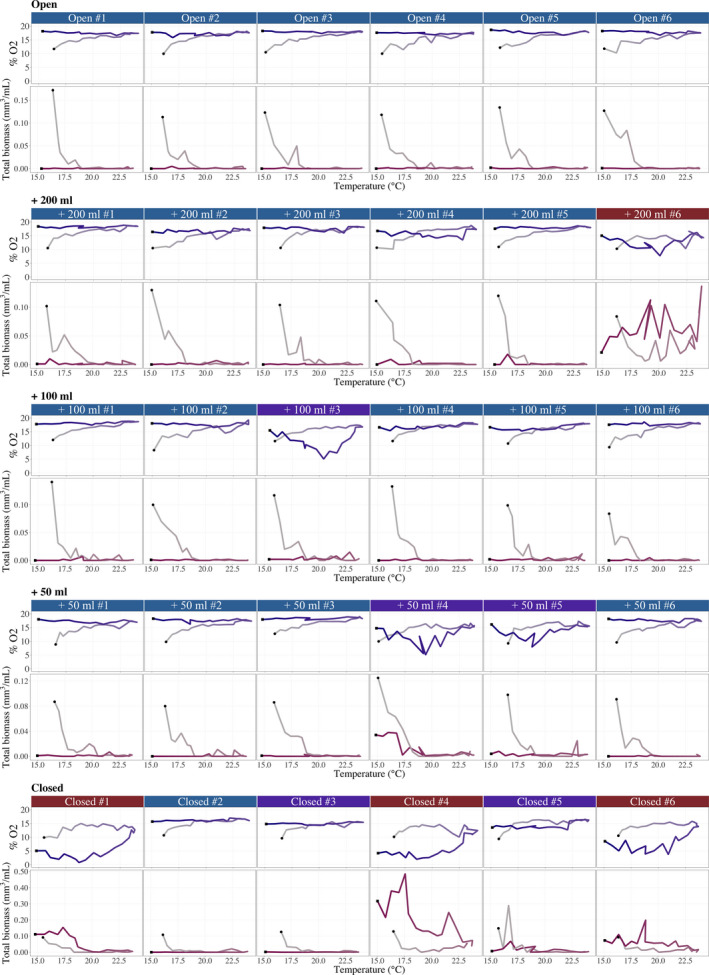
Ecosystem variables (DO and total biomass) across the temperature and temporal gradients. Each row shows a gas exchange treatment with six replicates. The color gradient represented the temporal change: from gray to blue (for DO) and from gray to violet (for total biomass). The label colors correspond to the clusters in Figure 5b

**FIGURE 7:**
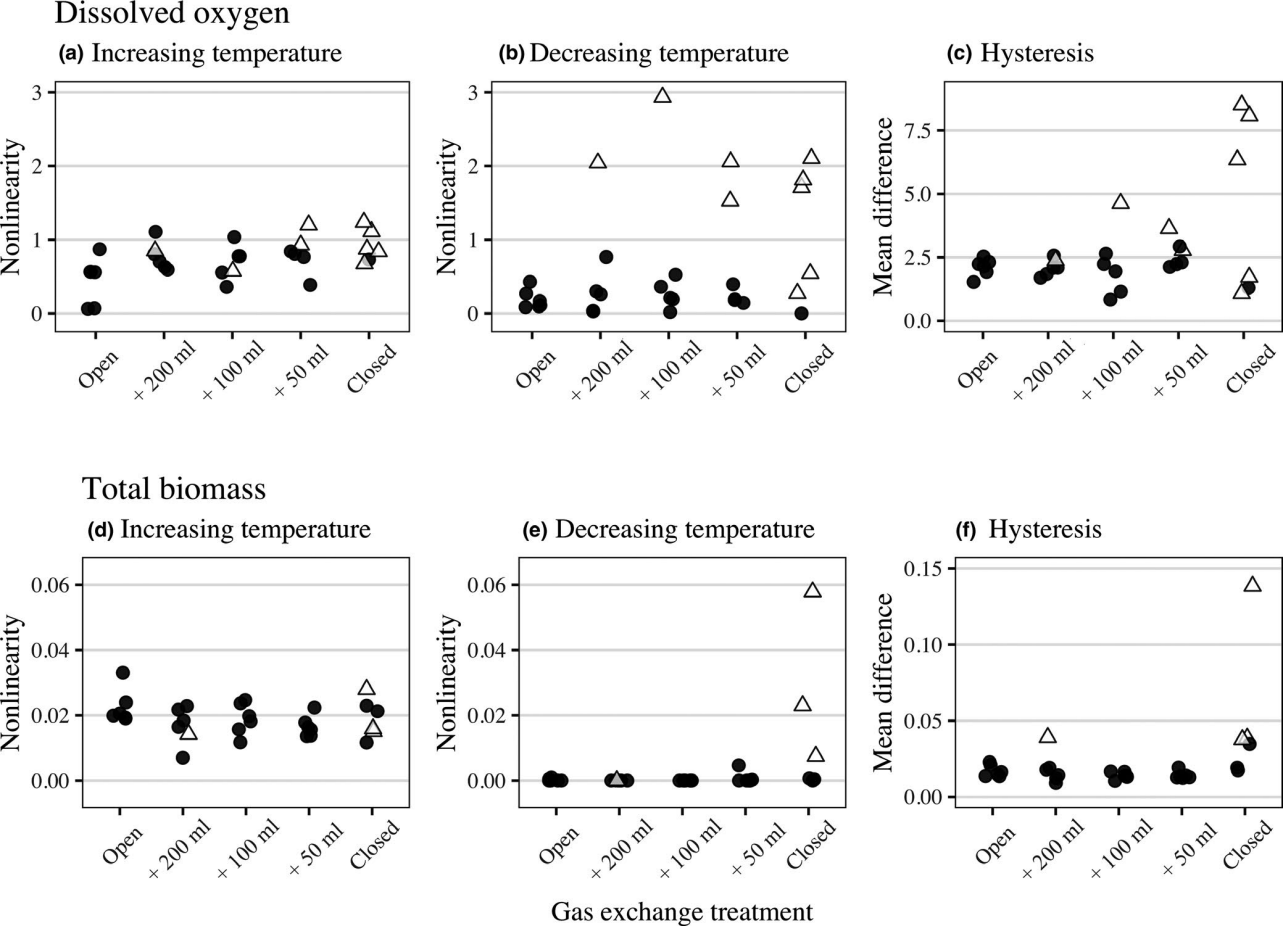
Effects of gas exchange treatment on nonlinearity and hysteresis of the ecosystem variables: (a‐c) DO and (d‐f) total biomass. The nonlinearity of the relationship between ecosystem variables and temperature is shown during the first half of the experiment when temperatures were increasing (a, d) and during the second half when they were decreasing (b, e). The measurement of hysteresis (mean absolute difference between paired measurements) for each microcosm is shown according to their gas exchange treatment (c, f). The pairing of measurements was made at the same temperature, one during the temperature increase, and the other during the temperature decrease. The symbols and colors represent the clustering for cluster 1 (black circle) and cluster 2 (white triangle), similarly to Figure 5d, 5e

Gas exchange treatments associated with stronger feedbacks tended to cause greater hysteresis (Table 1; Figure 6c,f), highlighting the greater divergence of total biomass between the increasing and decreasing temperature phases (i.e., hysteresis) with increasing feedback strength. For the hysteresis measured with DO dynamics (Figure 7c), the effect was found not significant due to considerable variability among replicates (Figure 6). However, the mean difference of DO varied significantly between the two clusters (Table 1), notably with a larger mean difference in the microcosms with the strongest feedback (Figure 7c in closed microcosms). For the hysteresis measured with total biomass dynamics (Figure 7f), both the gas exchange and the cluster affected the mean difference (Table 1).

To summarize, when the information of the clusters was not included, the effects of feedback strength were weaker for hysteresis and nonlinearity when the temperature decreased. Therefore, with changing environmental conditions, nonlinearity and hysteresis depend greatly on the system cluster/state.

## 4 DISCUSSION

Our experimental finding that manipulating the strength of the OEF by reducing gas exchange increased hysteresis and nonlinearity corresponds with the predictions of feedback systems theory. However, we did not observe any catastrophic shift between two ecosystem states (e.g., high and low levels of DO and total biomass), but rather a continuous transition (Figure 6). This result is coherent with a feedback strength *p* < 4 in a simple ecosystem model (Figure 3d). Continuous transitions are also observed in systems with weak positive feedback (Figures 1a and 2a; Kéfi et al., 2016; Scheffer et al., 2001) and stronger negative feedback (e.g., Clements & Ozgul, 2016, 2018). Therefore, our manipulation of feedback did not appear to make the ecological dynamics switch from those characteristics of a system dominated by negative feedback to those characteristics of a system dominated by positive feedback. We believe that the observed hysteresis results from mechanisms other than strong positive feedbacks, which create alternative stable states.

In communities with low hysteresis in DO, the predator–prey system appeared to become functionally extinct, while in communities with high observed hysteresis in DO, the predator–prey system persisted and consumed a significant amount of DO from the water. We can interpret these results in two ways. Firstly, in the presence of a positive feedback loop, the recovery of the predator–prey system could be explained by the mismatch between the rate of environmental change and the biological responses with an overly fast environmental change (Figure 3d) or a slowing down of the system dynamics. Secondly, in the absence of a positive feedback loop, the recovery could be due to a combination of two mechanisms: (a) the oxygen limiting the predation rate (Decker et al., 2004; Nestlerode & Diaz, 1998; Sandberg et al., 1996), which acted simultaneously with (b) the increasing temperature that potentially favored prey growth over predator growth on account of their different maximum growth rates (*r*_max_) scaled to their body size (i.e., the *r*_max_ of a large predator will occur at a lower temperature compared to that of a small prey at a higher temperature; Brown, Gillooly, Allen, Savage, & West, 2004b; Angilletta, Steury, & Sears, 2004). In both scenarios, the observed hysteresis appears to be only *apparent*, resulting from a transient state combined with the recovery of the predator–prey system impacting the total biomass and DO levels. Moreover, the absence of hysteresis resulted from the extinction of the biological system, driven by the temperature (Clements, Collen, Backburn, & Petchey, 2014; Long, Petchey, & Holt, 2007). Population cycles and extinctions are typical dynamics in predator–prey systems (Fussmann et al., 2000). In closed experimental systems (e.g., microcosm) in particular, extinctions are often observed due to the relatively homogeneous environment (e.g., no refuge for prey) (Hauzy, Tully, Spataro, Paul, & Arditi, 2010) and the potentially small population size (Pimm, Jones, & Diamond, 1988). Hence, the difference in population dynamics observed within treatments could result from chance alone, which would then have large effects on subsequent system dynamics.

What do we mean by *apparent hysteresis*? Hysteresis is the difference in the trajectory of the ecosystem state with different directions of environmental change. The observation of changes in hysteresis alongside changes in OEF strength is only indicative of alternative stable states if the system dynamics are relatively slow compared to the environmental change. In addition to emphasizing the importance of considering changes in the system timescale that accompany changes in the feedback, our findings point to the benefits of studies that aim to test the underlying assumptions or mechanisms of theories about how ecosystems respond to environmental change. It is *apparent*, because the system *appears* to exhibit hysteresis. A different measure of hysteresis, and specifically one that assumes or requires very slow environmental change to reach equilibrium, would not show evidence of hysteresis in the absence of alternative stable states. Some believe that this different measure should be termed *real hysteresis*, while others may claim that *apparent hysteresis* does not exist, because from a certain perspective, it may appear to be internally inconsistent. Indeed, the difference between *apparent* and *real* hysteresis is that if a system only displays *apparent* hysteresis, then it is only a matter of time before the ecosystem returns to its previous state, whereas a system with *real hysteresis* will never return. This *apparent* hysteresis relates to debates about stable versus transient states (e.g., Fukami & Nakajima, 2011; Hastings, 2004). Indeed, natural communities may rarely reach stable states due to the mismatch between community response to disturbance and the disturbance regime (Pickett & White, 1985). These points to the need for a more careful consideration of the rate of environmental driver change relative to the rate of system change and their combination with changes in the strength of OEF.

We did not assess the stability of the observed ecosystem states. Yet the predictions of the tested theory on nonlinearity and hysteresis assume that the equilibria are locally stable. Hence, without a deeper mechanistic understanding of the system perhaps aided by a parameterized mechanistic model, we could not confirm that the match between experimental and predicted treatment effects occurred for the right reasons (i.e., difference in timescale); this could therefore be a coincidence. Similarly, we could not ascertain the stability of the clusters for the community time series observed; thus, we could not test for the effects of the feedback treatment levels on the likelihood of alternative stable states. A longer experiment and/or different temperature variation treatments may provide information to support the presence of alternative regimes in community composition (Faassen et al., 2015; Siteur et al., 2014; Vanselow, Wieczorek, & Feudel, 2019). Another option would be to manipulate the initial conditions among the replicates and test for the effects of feedback strength on the modality of the relationship between the initial conditions and the long‐term community composition and dynamics (Dai et al., 2012).

With our manipulation of OEF strength, oxygen gained the potential to be the limiting resource in our predator–prey system. However, we lack quantitative information about organism responses to DO concentration (i.e., for the growth rate variation with this environmental variable, or whether it is linear). Furthermore, the different species of microbes in our experiment may exhibit different responses to DO concentration. For example, some species of protozoa thrive in anoxic conditions, while others are more common in the presence of oxygen (Bick, 1973). *Colpidium striatum* and *Serratia fonticola* can grow either in aerobic or anaerobic conditions (Fenchel, 2005); it is unknown whether *Spathidium* is affected by low oxygen levels (Andrushchyshyn, Magnusson, & Williams, 2003). Again, recording the responses of individual species to DO concentration across temperatures in a factorial design would provide some of the information required to better understand the mechanistic causes of the patterns observed in our experiment, potentially by constructing a model for the joint dynamics of species biomass and oxygen flux. A further question about the experimental system relates to whether the prey or predator benefits in ways other than increased productivity caused by the presence of wheat seeds. We are unaware of any such benefit. While some other benefits have so far been undiscovered, wheat seeds were present in all treatments, and so such a benefit would be independent of any treatment, although we cannot rule out the possibility that such a benefit could interact with the treatment.

Overall, a great deal of research remains to be done. Firstly, a theoretical model of our system, ideally parameterized with empirical data about how organisms affect their environment and vice versa, would allow us to confirm that we are observing the predicted patterns (i.e., greater nonlinearity and hysteresis) for the right reasons. Secondly, it is a priority to understand the importance of OEF strength given that there are various environmental changes other than temperature and that multiple simultaneous changes occur. Thirdly, the effects of biodiversity on the influence of OEFs are important in order to understand and predict species richness and composition, which may both drive and respond to environmental change. Finally, we imagine that novel empirical manipulations of OEF strength, such as our own, have the considerable potential to shed light on the processes governing ecological dynamics: for example, how gradual environmental change might result in abrupt ecological changes (Ratajczak et al., 2018).

As pointed out by Scheffer and Carpenter (2003), although observations can provide hints, experiments, while limited in scale and realism, are an essential element of any research on the existence of alternative attractors. Presumably, the importance of experiments should apply equally, if not more, when examining the features of ecosystems in which nonlinearities, hysteresis, and alternative stable states are more or less likely. Despite the importance of theories about feedbacks, nonlinearity, hysteresis, and multistability for understanding ecosystem dynamics, we could find no previous studies manipulating OEF strength. As our study raises many questions and shows that inference can be complicated since OEF manipulations can also affect the timescale of the system dynamics, we hope that this leads to a refinement of the experimental methods used for testing the mechanism responsible for determining how ecosystems respond to environmental change.

## CONFLICT OF INTEREST

We declare that we have no competing interest.

## AUTHOR CONTRIBUTION

**Aurélie Garnier:** Conceptualization (equal); Data curation (lead); Formal analysis (equal); Investigation (equal); Methodology (equal); Project administration (equal); Visualization (lead); Writing‐original draft (lead); Writing‐review & editing (equal). **Florence Hulot:** Formal analysis (supporting); Methodology (equal); Supervision (supporting); Validation (supporting); Visualization (supporting), Writing‐review & editing (equal). **Owen Petchey:** Conceptualization (equal); Formal analysis (equal); Funding acquisition (lead); Investigation (equal); Methodology (equal); Project administration (equal); Resources (lead); Supervision (lead); Validation (lead); Visualization (supporting); Writing‐review & editing (equal).

## Data Availability

Should the manuscript be accepted, the data supporting the results and the reproducible reports containing all the analyses, figures, and table will be available at http://github.com/garnier‐aurelie/OEF‐strength and archived with doi on Zenodo.
